# PO-YOLOv5: A defect detection model for solenoid connector based on YOLOv5

**DOI:** 10.1371/journal.pone.0297059

**Published:** 2024-01-26

**Authors:** Ming Chen, Yuqing Liu, Xing Wei, Zichen Zhang, Oleg Gaidai, Hengshou Sui, Bin Li

**Affiliations:** 1 College of Engineering Science and Technology, Shanghai Ocean University, Shanghai, China; 2 CNFC Overseas Fisheries Co., Ltd., Beijing, China; University of Baghdad, IRAQ

## Abstract

Solenoid connectors play important role in electronic stability system design, with the features of small size, low cost, fast response time and high reliability. The main production process challenge for solenoid connectors is the accurate detection of defects, which is closely related to safe driving. Both faultless and defective products have similar color and shape at the defect location, making proper inspection challenging. To address these issues, we proposed a defect detection model called PO-YOLOv5 to achieve accurate defect detection for solenoid connectors. First, an additional prediction head was added to enable the model to acquire more semantic information to detect larger-scale defective features. Second, we introduced dynamic convolution to learn complementary connections between the four dimensions of the convolution kernel by utilizing its multidimensional attention mechanism. Replacing conventional convolution with dynamic convolution enhances the detection accuracy of the model and reduces the inference time. Finally, we validated PO-YOLOv5 versus the state-of-the-art object detection methods on the same solenoid connectors dataset. Experiments revealed that our proposed approach exhibited higher accuracy. The mAP (mean Average Precision) result of PO-YOLOv5 was found to be about 90.1%. Compared with the original YOLOv5, PO-YOLOv5 exhibited improved precision by about 3%.

## Introduction

With the rapid development of driverless technology, the demand for electronic stability system in safe driving is increasing. As a crucial component of the electronic stability system, the solenoid connector relies on strong electromagnetic forces to move solenoid core and regulate pressure in the wheel brake cylinders. It makes braking safer and more reliable by converting braking signal into a high-frequency control signal. However, manufacturing process complexity and instability of the soldering process inevitably lead to product defects, which may seriously affect safe vehicle operation. Therefore, it is important to identify these defective solenoid connectors in time. At present, high-quality test result heavily relies on experienced inspectors, which is time consuming and costly. Additionally, the probability of manual misdetection is not negligible. Thus, there is a need for high-precision automatic inspection methods that can replace manual inspection.

**Fig 1:**
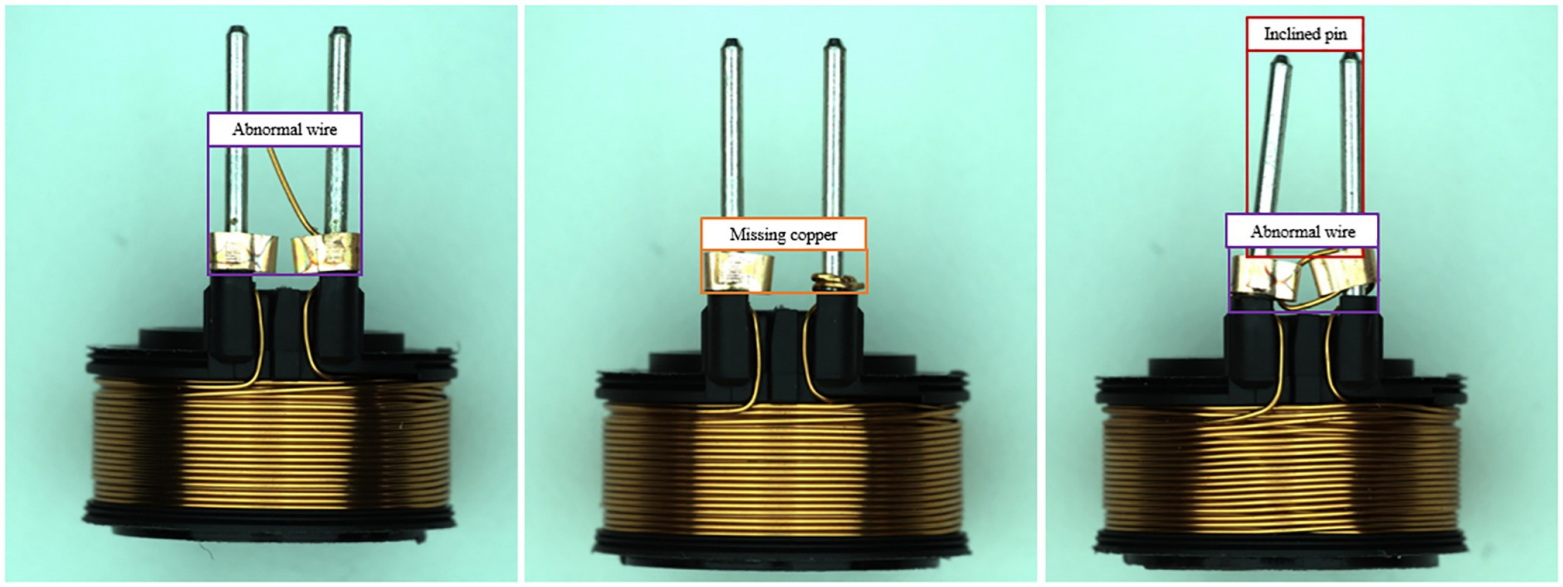
Common defects in the production of solenoid connectors, including missing copper (indicated by the orange box), abnormal wire (indicated by the purple box) and Inclined pin (indicated by the red box).

Generally, three types of defects can occur in solenoid connectors during manufacturing process: Missing copper (Mc), Abnormal wire (Aw) and Inclined pin (Ip), as shown in Fig 1. Two problems are frequently discussed during automatic defect inspection of solenoid connectors: defect classification and defect location. However, the inspection methods need to maintain a high level of accuracy to correctly classify and locate defects, which relies on computing equipment and long detection time. Actual production inspection tasks executed on a computing constrained platform require fast detection speed and high detection accuracy. Therefore, it is critical to improve classification and positioning accuracy of the detection, while ensuring real-time detection of defects in solenoid connectors.

Traditional machine vision methods rely on feature extractors manually designed by experts to classify and locate defects [1, 2], such as SIFT (Scale Invariant Feature Transform) [3], HOG (Histogram Oriented Gradient) [4], LBP (Local Binary Pattern) [5] and SVM (support vector machines) [6]. Zhai et al. [7] proposed a method that uses a Kalman filter with constant velocity model to filter the images and then uses the measured residuals obtained from the Kalman filter to detect aluminum foil defects. An adaptive multiscale geometric analysis method named RNAMlet was proposed to identify surface defects of steel [8], which adapts its own computational cost according to the complexity of the image. Liu et al. [9] constructed a new feature extraction method called NSST-KSR, which can effectively improve the recognition of surface defects in aluminum sheets. It combines both nonsubsampled shearlet transform (NSST) and kernel spectral regression (KSR) to extract features more efficiently, and then uses SVM for classification. Li et al. [10] proposed a classification algorithm based on LBP and local binary differential excitation pattern (LB_DEP) to extract birch surface defect features. The LB_DEP is a texture description model, which was generated using combination of LBP and Weber’s Law. These manually designed features depend on the type of defect, making traditional machine vision methods highly interpretable. However, due to the sensitivity to environmental factors such as lightning conditions, traditional machine vision methods require complex thresholds and different feature extraction methods for different detection tasks, making them unsuitable as a universal detection method.

Due to incremental breakthroughs in computing hardware performance and rapid development of deep learning, object detection networks are gradually being applied to industrial defect detection [11]. Compared with classical machine vision methods, deep learning methods have superior robustness. Neural networks can learn features directly from images and have higher representation ability for complex semantic information. It replaces hand-designed features with an end-to-end approach. According to whether region proposal is required, the object detection models based on deep learning can be classified into two categories: one-stage detectors and two-stage detectors.

The two-stage detector generates region proposals based on the extracted features and then performs a secondary correction for the region proposals to obtain the detection results, which are characterized by high computation and high detection accuracy. Common two-stage detectors include R-CNN [12], Fast R-CNN [13] and Faster R-CNN [14]. A two-stage network consisting of a segmentation network and a decision network was proposed to detect surface abnormal defects [15]. The proposed approach first performs a pixel-wise localization of the surface defect by the segmentation network followed by a decision network for the classification of the binary image. The results showed that the proposed method outperformed other methods at that time in the field of surface crack detection. Su et al. [16] proposed an end-to-end faster RPAN-CNN detection framework which forms more accurate defect region proposals by embedding a complementary attention network (CAN) into a region proposal network (RPN). The RPAN-CNN framework largely improved detection accuracy in the field of solar cell defect detection. Wei and Bi. [17] introduced the feature pyramid network (FPN) into the Faster R -CNN to improve the network’s ability to detect multi-scale defects. Liu et al. [18] proposed a multiscale context detection network, MSC-DNet, based on Faster R-CNN to detect surface defects in steel strips. A parallel architecture with different dilation rates of dilation convolution (PADC) is established to capture the contextual information of multiscale defects. These two-stage detection methods can meet the needs of industrial end-to-end detection with high detection accuracy. However, due to the complexity of the two-stage network structure, these methods require large computational resources and longer inference time to complete the task.

One-stage detectors primarily use regression to directly extract features from the network to predict the classification and location of the object, such as SSD [19] and YOLO series [20–23]. Compared to two-stage detectors, one-stage detectors save a lot of time at the expense of a little accuracy. In practical quality detection, the system needs to detect defects of different types and scales in real-time, and one-stage detectors can achieve the above-mentioned requirements. Kou et al. [24] proposed an end-to-end defect detection model based on YOLOv3 to effectively improves the detection accuracy of defects such as pitted surface, scratch and crazing cracks. It reduces the computation time by replacing the anchor-based feature selection mechanism with anchor-free, and introduces dense convolutional blocks to enhance the network feature extraction ability. Ma et al. [25] proposed a lightweight detection method based on YOLOv4 for surface defects on aluminum strips, which utilizes a depthwise separable convolutional reconstruction backbone network with embedded two-channel attention module and SPP module to improve the detection accuracy of the model for multi-scale objects. The result shows that the proposed method achieves a better trade-off between speed and accuracy. Based on YOLOv4, Li et al. [26] modified the three techniques of channel attention, multi-space pyramid pooling, and EMA to detect defects in wire and arc additive manufacturing, obtaining a 94.5% recognition rate. A new detection model GBH-YOLOv5 was proposed for detecting Photovoltaic panel surface defects [27], which adds a prediction head for better detection of small defects and utilizes Ghost convolution to improve the model inference speed. Jin et al. [28] embedded the GSConv and CBAM modules into YOLOv5 to detect both Gap and Glueless defects among the glass wool dataset. Hu et al. [29] optimized the YOLOv5 model by integrating the CBAM module and modifying the loss function, and then applied it to intelligent detection of citrus epidermal defects.

As one of the most popular object detection methods in recent years, YOLOv5 achieves both accuracy and efficiency with low deployment difficulty. It is suitable for engineering applications. However, there are two problems when applying the one-stage object detection model to the solenoid connector dataset. First, conventional defect detection networks only detect a particular anomalous object. It is necessary to discern the degree of defects based on the relative position relationship of objects when detecting solenoid connectors. This brings enormous difficulties to the network. Second, the similarity in color and shape of the Missing copper (Mc) to the Abnormal wire (Aw) can easily lead to misjudgment. The above-mentioned networks do not specifically optimize YOLOv5 for defects which contain relative positional information in solenoid connectors. In order to adapt it to the task of detecting defects in solenoid connectors, we have specifically optimized the network structure of YOLOv5.

In this paper, we proposed an improved model, named PO-YOLOv5 to address the above-mentioned problems. In the Neck, we introduced an extra prediction head to detect defects containing relative position information. PO-YOLOv5 can extract more semantic information because it has 64 down-sample layers. Then, by replacing conventional convolution with Omni-dimensional Dynamic Convolution (ODConv) [30] in the Neck, we changed the conventional convolutional concept of treating data equally, so that each input sample has its own convolutional weights which improved model expressiveness. Moreover, ODConv also significantly improved detection accuracy of the model with almost no increase in the parameters of model.

The main contributions of our work are summarized as follows:

- A new prediction head was added to extract more semantic information, and the experiments prove that the improved network effectively improves the detection accuracy of the model.
- By replacing part of the conventional convolution of the Neck with the ODConv module, the model’s feature learning and extraction capability are greatly enhanced.
- Unlike conventional annotation methods that only annotate the specific location of the defect, we proposed an annotation method based on relative position information, which has been demonstrated to have superior detection accuracy on the solenoid connectors dataset.

## Materials and methods

### System overview

**Fig 2:**
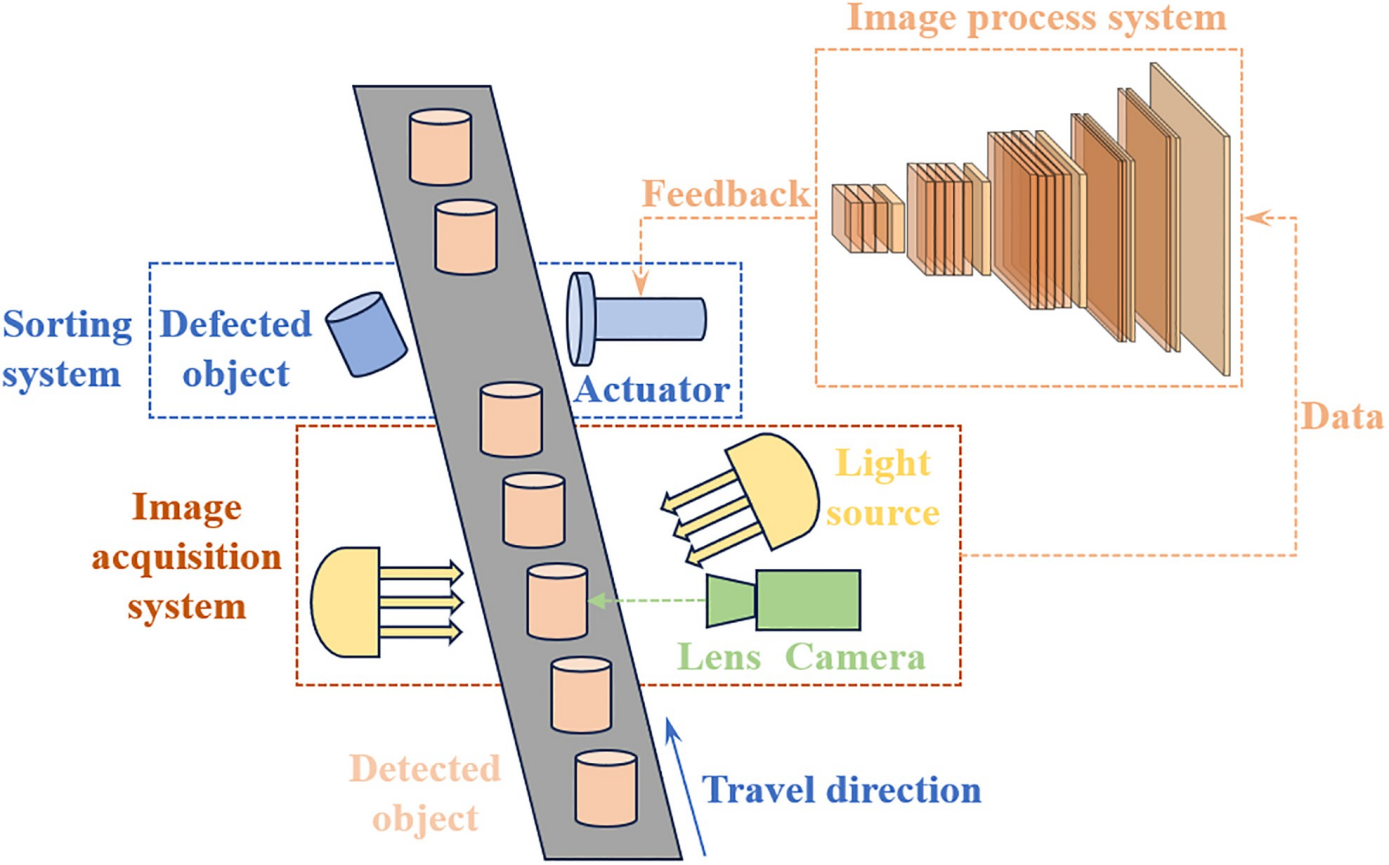
Schematic diagram of defect detection system.

In this research, the solenoid connectors images are captured and processed using the following visual defect detection system, which consists of three components: image acquisition system, image processing system, and sorting system. The schematic diagram of the solenoid connector defect detection system is shown in Fig 2. The data acquired from the image acquisition system is transferred to the YOLOv5 model for defect detection. When a defective product is detected, the system stops the conveyor and controls the actuator to separate the defective product.

To achieve accurate and fast acquisition of solenoid connector defect images, we built a solenoid connector defect image acquisition system, which mainly consists of three parts: industrial camera, lens and light source. At first, it is necessary to avoid reflection when selecting the light source because of the metal surface of the solenoid. After extensive testing, a square backlight light source was finally selected. Furthermore, as the input of model, the image quality determines the quality of the model to a great extent. We used a 5MP color aerial camera with an 8 MP lens to obtain clear images of the solenoid connectors and then applied these images to construct the solenoid connector dataset.

### Data augmentation

Datasets in industrial manufacturing usually contain sparse images, which means that data augmentation is needed to expand the dataset. In addition, the detection performance of the model is closely related to the batch size during training. Setting a larger batch size means that the network computes more images at a time, thus reducing the training time and improving the stability of the network. However, a large batch size means a high GPU occupancy, which conflicts with the requirements for hardware devices in industrial applications.

**Fig 3:**
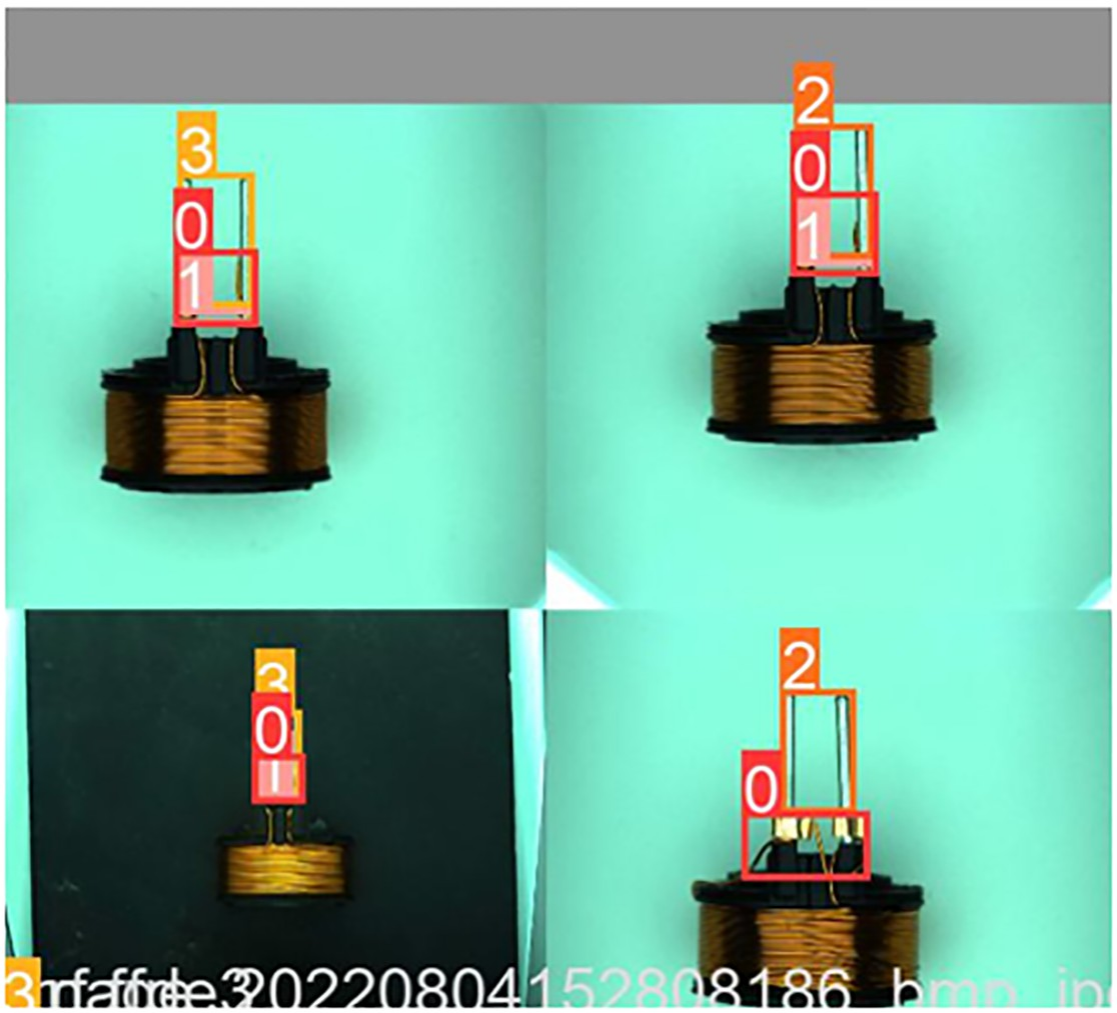
Examples of mosaic data augmentation.

To address the above issues, we use mosaic data augmentation method to enhance the robustness of the model. Mosaic data augmentation randomly crops and splits the four training images into a single image, significantly enriching the dataset and enhancing the robustness of the model. Fig 3 shows the effect after the mosaic data augmentation. Merging four images into one and feeding them into the model is equivalent to indirectly increasing the batch size. That reduces the occupation of GPU and decreases the hardware requirements.

### Overview of YOLOv5

The basic YOLOv5 network is divided into four versions according to width and depth, namely YOLOv5s, YOLOv5m, YOLOv5l and YOLOv5x. To ensure the speed of product detection, YOLOv5s is chosen as our baseline.

**Fig 4:**
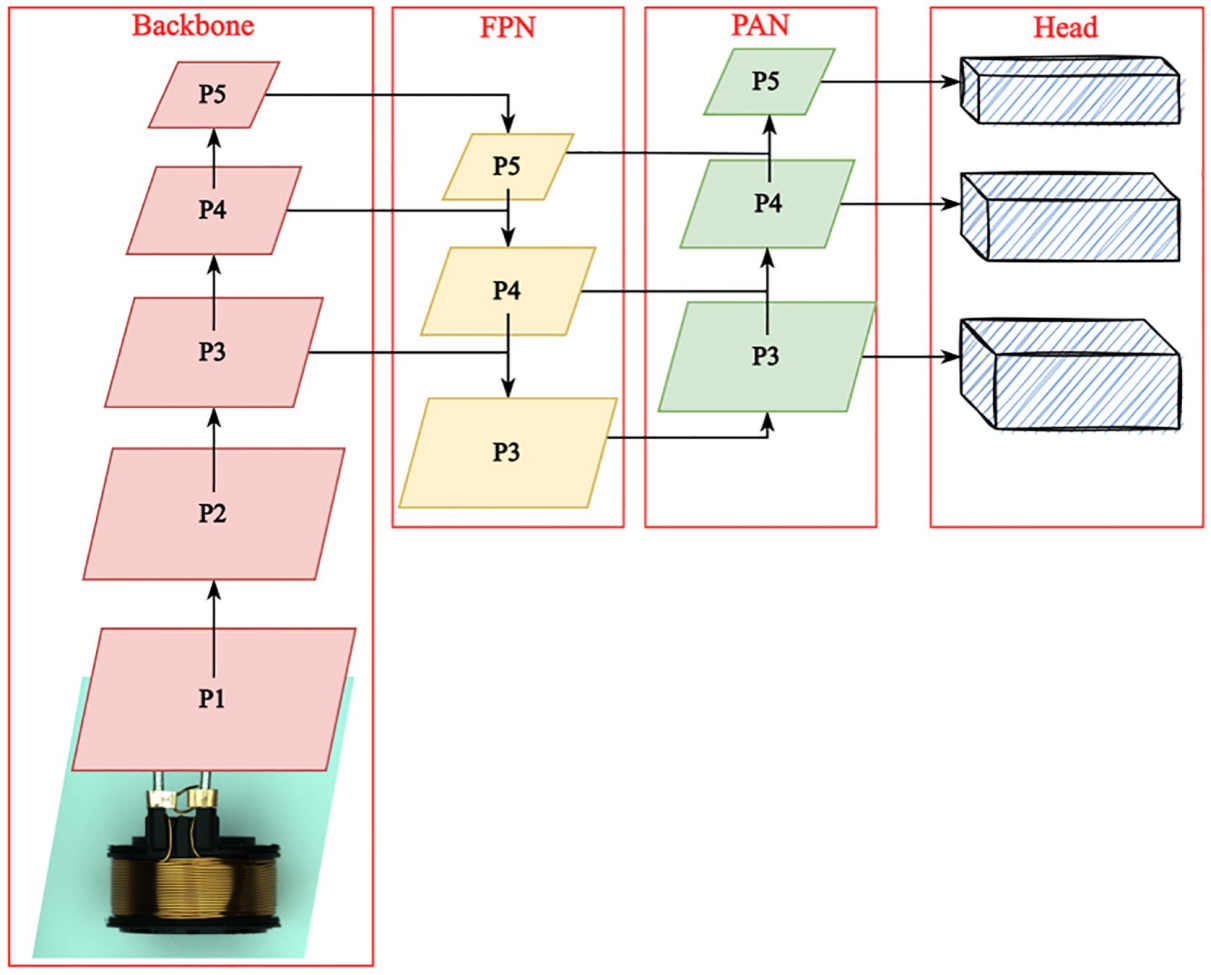
The architecture of the PO-YOLOv5. (a) CSPDarknet53 backbone. (b)Four ODConv modules were added in the Neck. In addition, the number of each block is marked with orange numbers on the left side of the block.

YOLOv5 mainly consists of three parts: Backbone, Neck and Head. As shown in Fig 4. After data augmentation, the input image data are sent to Backbone, which performs feature extraction through the CSPDarknet53 [31]. FPN and Path Aggregation Network (PANet) [32] are used to aggregate the image feature at this stage in the Neck. Finally, the network performs target prediction and output in the Head.

### PO-YOLOv5

**Fig 5:**
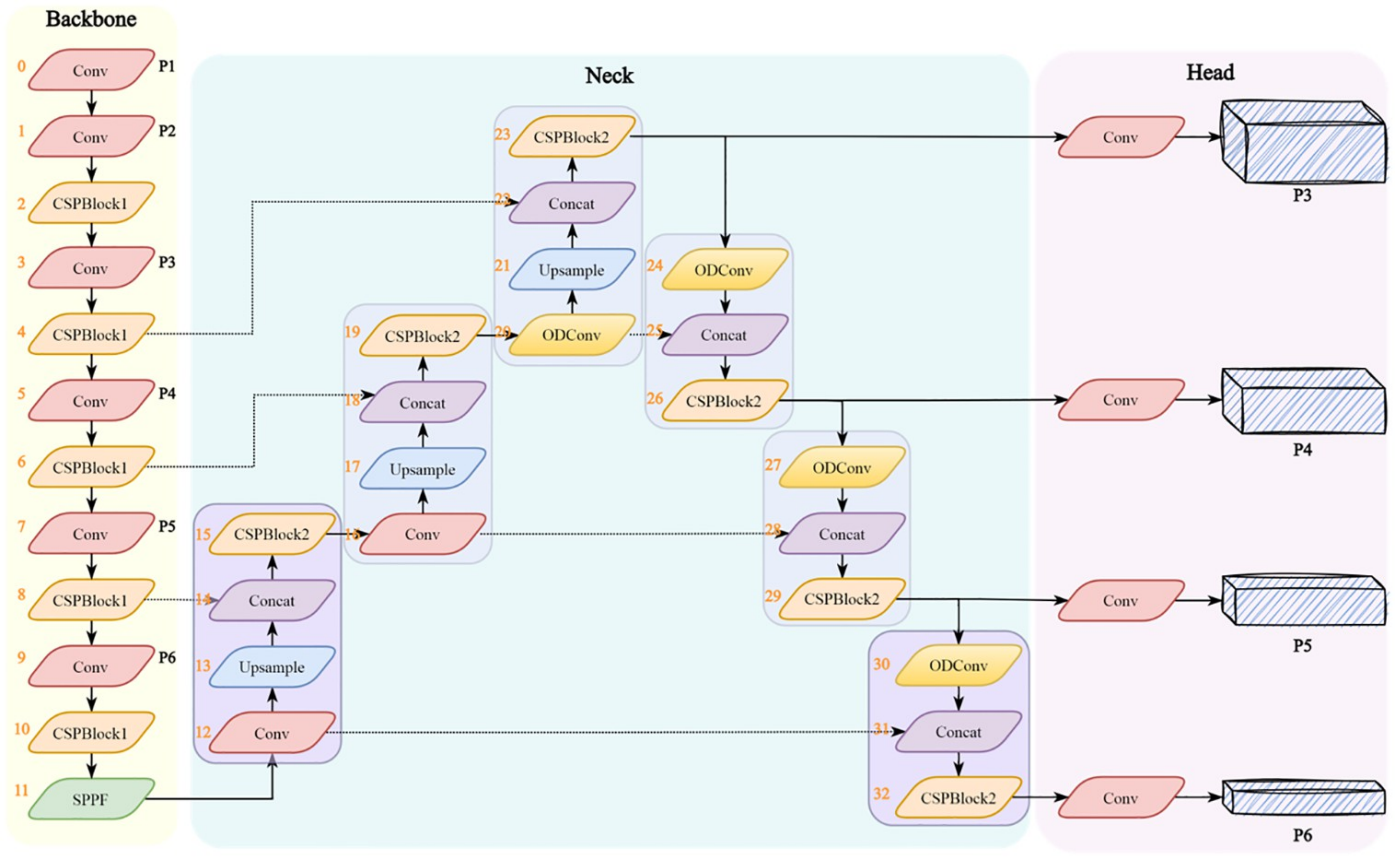
Physical diagram of the device of image acquisition. 1. Industrial camera, 2. Lens, 3. White coaxial light source, 4. Square backlight source, 5. Digital controller.

We modified the original YOLOv5 to make it specialize in the Solenoid Connectors dataset. The framework of PO-YOLOv5 is illustrated in Fig 5.

#### Prediction head for relative position information

We investigated the Solenoid Connectors dataset and found that various defects containing relative position information, such as copper sleeves placed over the pins, and the two pins normally being held in a parallel position. Therefore, we added a prediction head to enhance the ability of detecting relative location information of defects. As shown in Fig 5, the added prediction head is generated from the high-level, low-resolution feature maps, which are more sensitive to defects containing relative position information. Combined with the other three prediction heads, our four-head structure can detect defects at different scales. Although computational and memory costs increased after adding a prediction head, the performance of defect detection is greatly improved.

#### ODConv module

**Fig 6:**
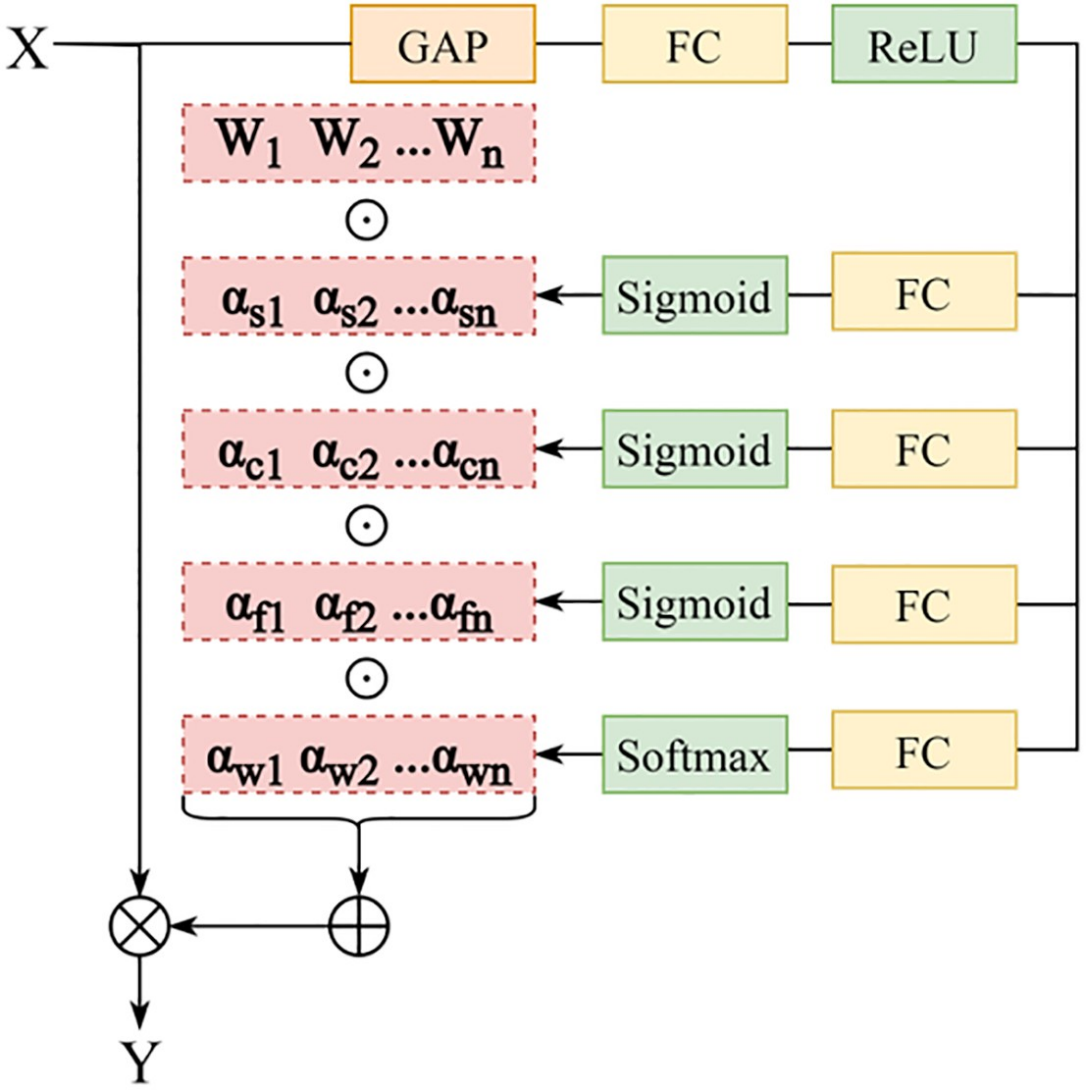
ODConv module. Convolutional kernel space consists of the spatial kernel size, the input channel number, the output channel number and the convolutional kernel number.

Using a single static convolutional kernel in each convolutional layer is the common training paradigm of modern Convolutional Neural Networks(CNN) [30]. Static convolution uses the same convolution kernel to perform the same sliding window calculation for all input images, while dynamic convolution adjusts the input image accordingly, adding a dynamic update process to the convolution parameters [33]. Inspired by the Omni-Dimensional Dynamic Convolution, we replace some static convolutional blocks in the Neck with ODConv modules to strengthen the feature extraction ability of model and make an effective trade-off between recognition speed and accuracy. The structure is shown in Fig 6. ODConv modules leverage a multi-dimensional attention mechanism with a parallel strategy to learn four types of attentions for convolutional kernels along all four dimensions of the kernel space [30]. Mathematically, ODConv can be defined as

$$\begin{array}{c} y=(\alpha_{w1}⊙\alpha_{f1}⊙\alpha_{c1}⊙\alpha_{s1}⊙W_{1}+\ldots +\alpha_{wn}⊙\alpha_{fn}⊙\alpha_{cn}⊙\alpha_{sn}⊙W_{n})*x \end{array}$$ (1)

Where $x\in R^{h\times w\times c_{in}}$ and $y\in R^{h\times w\times c_{\text{out}\;}}$ denote the input features and the output features; $\alpha_{wi}\in R$, $\alpha_{si}\in R^{k\times k}$, $\alpha_{ci}\in R^{c_{in}}$ and $\alpha_{fi}\in R^{c_{\text{out}\;}}$ denotes the attention scalar along the four dimensions of the number of convolutional kernels, spatial dimension, input channel dimension, and output channel dimension of the convolutional kernel *W*_*i*_; ⊙ denotes the multiplication operations along different dimensions of the kernel space.

The input x is squeezed into a feature vector of size *c*_*in*_×1×1 by channel-wise Global Average Pooling (GAP) operations first. Subsequently, the Fully Connected (FC) layer maps the squeezed feature vector to a lower dimensional space to avoid high model complexity. It is divided into four branches after being nonlinearly activated by the Rectified Linear Unit (ReLU) [34]. Each branch has an FC layer with the output size of k×k, *c*_*in*_×1, *c*_*out*_×1 and n×1, and Softmax or Sigmoid functions were used to generate the normalized attentions *α*_si_, *α*_ci_, *α*_fi_ and *α*_wi_.

Applying dynamic convolution to numerous convolutional layers will severely increase the model size [33], causing difficulties in training due to excessive computation. In addition, dynamic convolution brings significantly higher improvements in the deeper layers of the network than the shallow layers [30]. Therefore, we only applied the ODConv module in the Neck based on the original YOLOv5.

## Experiment and analysis

### Dataset

**Fig 7:**
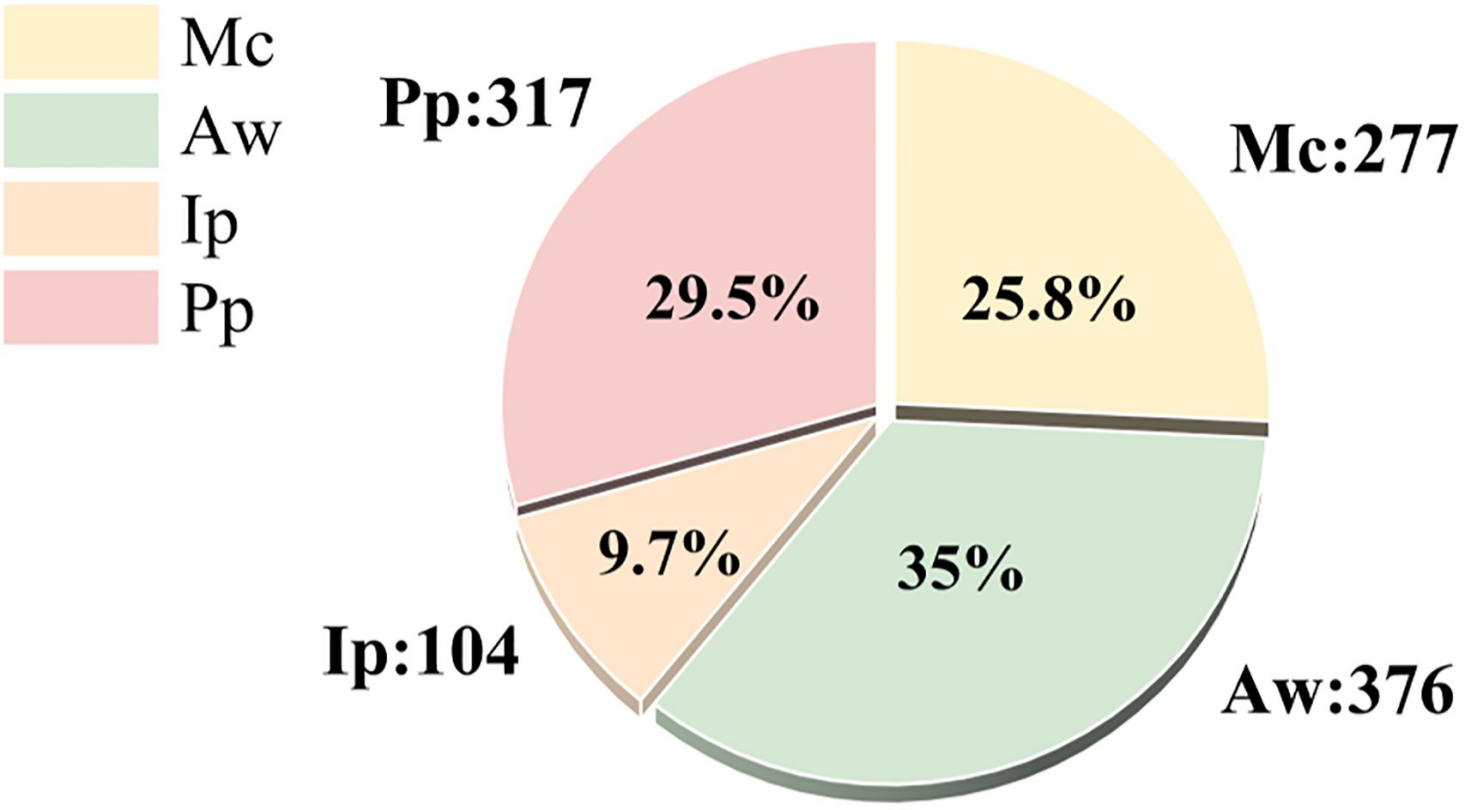
Distribution of four types of detection categories from the solenoid connectors dataset.

In this section, we evaluated the improved model performance of YOLOv5 with the self-made solenoid connectors dataset, which includes four types of detection categories. They are Missing copper (Mc), Abnormal wire (Aw), Inclined pin (Ip) and Parallel pin (Pp). Since the inclined pins have high similarity to the parallel pins and CNN has difficulty in extracting relative position information, we also labeled the parallel pin to enable the network to acquire more prior information while preventing misclassification. The distribution of four types of detection categories in the dataset is shown in Fig 7.

### Experimental setup

We used the PyTorch 1.12.0 [35] to implement all experiments on an NVIDIA RTX 3070 graphics card. The training and validation datasets include 421 images and the test dataset includes 47 images. Since PO-YOLOv5 downsamples twice as much as the original YOLOv5, we resized the input image from 640×640 to 1280×1280 to prevent difficulties in extracting features due to the low resolution of the feature map.

During the training process, we used the standard SGD optimizer with decay and momentum of 0.937 to train all the models. The training process makes use of a warm-up strategy, learning rate decay [36], L2 regularization [37], and data preprocessing techniques [38]. The maximum rate of learning is 0.01, which is gradually decreased. Each network will undergo 600 epochs of training.

### Data analysis

**Fig 8:**
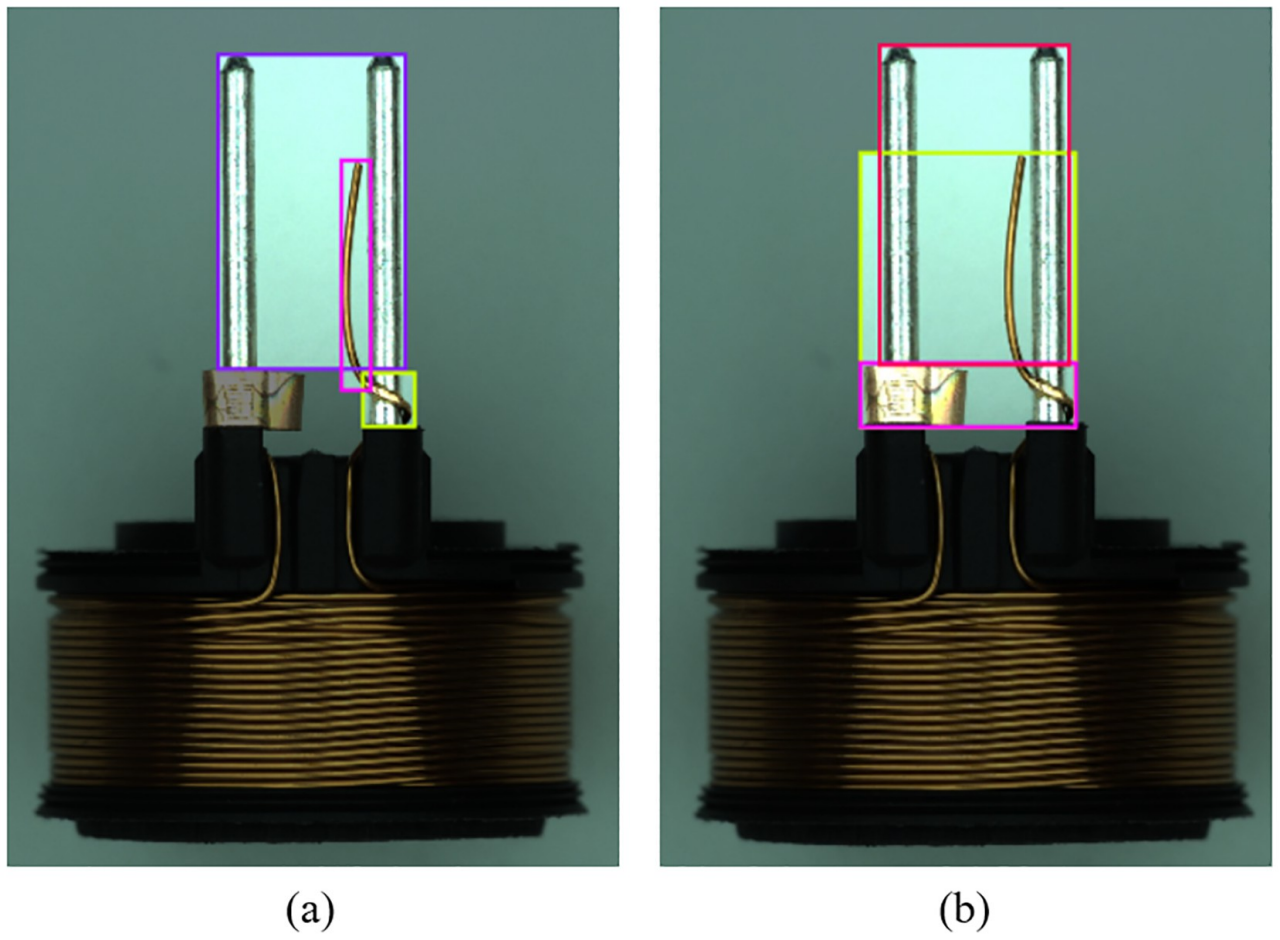
(a) Example of data annotation with only specific location annotated. (b) Example of data annotation with relative location information.

According to our previous engineering experience, the quality of the dataset annotation affects the accuracy of the final recognition and the precision of the positioning. We tested two types of annotation. Like most dataset annotation methods, the first annotation method only annotates the specific location of the defect, while the second annotation method contains the relative location information of the defect area, for example, the abnormal wire is usually wrapped around the pins and copper sleeves are soldered in pairs to the pins. As shown in Fig 8. Table 1 shows the detection results of two different annotation methods based on the PO-YOLOv5 algorithm. Through comparison, we found that the second labeling method was more accurate. We believe that the second annotation method allows deep neural networks to learn more semantic information and enhances the model’s ability to distinguish between foreground and background, thus achieving precise localization of defects.

**Table 1:** Comparison of two different annotation methods.

Annotation Method	Aw	Mc	Ip	Pp	all
Specific location annotation	65.0%	52.3%	86.1%	97.2%	75.3%
Relative Location annotation	**91.1%**	**84.1%**	**86.5%**	**97.8%**	**90.1%**

### Experimental analysis

To demonstrate the advantages of our proposed method on the Solenoid Connectors dataset, we compared it with the original YOLOv5, YOLOv4 [23], YOLOv6 [39], and YOLOv7 [40]. The evaluation metrics include model size, parameters, floating-point operations per second (FLOPs), mean average precision (mAP) and frames per second (FPS). The higher mAP and FPS represent the better detection performance of the model. The specific experimental results are shown in Table 2.

**Table 2:** Comparison of our method with other methods on the solenoid connectors dataset.

Method	Model	Parameters	FLOPs	mAP@0.5	mAP@0.5:0.95	FPS
YOLOv4	256.3M	52.53M	119.83G	80.6%	72.1%	55
YOLOv5s	14.4M	7.02M	15.80G	95.4%	87.1%	133
YOLOv6s	38.0M	17.19M	44.07G	93.7%	87.7%	114
YOLOv7	74.8M	37.21M	105.20G	95.1%	89.1%	89
Ours	25.8M	12.38M	**14.90G**	**96.8%**	**90.1%**	63

First, it can be seen from Table 3 that the metrics of YOLOv4 are not superior. Compared with YOLOv5, although YOLOv6 and YOLOv7 have increased in map@0.5:0.95, it is difficult to deploy on industrial production lines with inferior hardware equipment as the parameters and computation of the model are too large. Considering the detection speed and accuracy, we finally chose YOLOv5 as the benchmark for the experiment. Secondly, the model size and the number of parameters of our proposed method are 25.8M and 12.38M, which are second only to YOLOv5s. The FLOPs is only 14.90G. It can be seen from the above metrics that our method requires fewer hardware devices. In addition, our method achieves 90.1% mAP@0.5:0.95 on the Solenoid Connectors dataset, which is significantly higher than other methods. Finally, FPS is used as a measure of object detection speed, illustrating that our method is able to meet the requirements of real-time detection in the industry.

**Table 3:** Ablation study on the solenoid connectors dataset.

Method	Model	Parameters	FLOPs	FPS	mAP@0.5:0.95
YOLOv5s	14.4M	7.021M	15.8G	133	87.1%
YOLOv5s+ODConv	14.5M	7.052M	14.8G	122	87.6%(↑0.5)
YOLOv5s+P6	25.7M	12.320M	16.2G	64	88.5%(↑1.4)
PO-YOLOv5	25.8M	12.380M	14.9G	63	**90.1%**(↑3.0)

**Fig 9:**
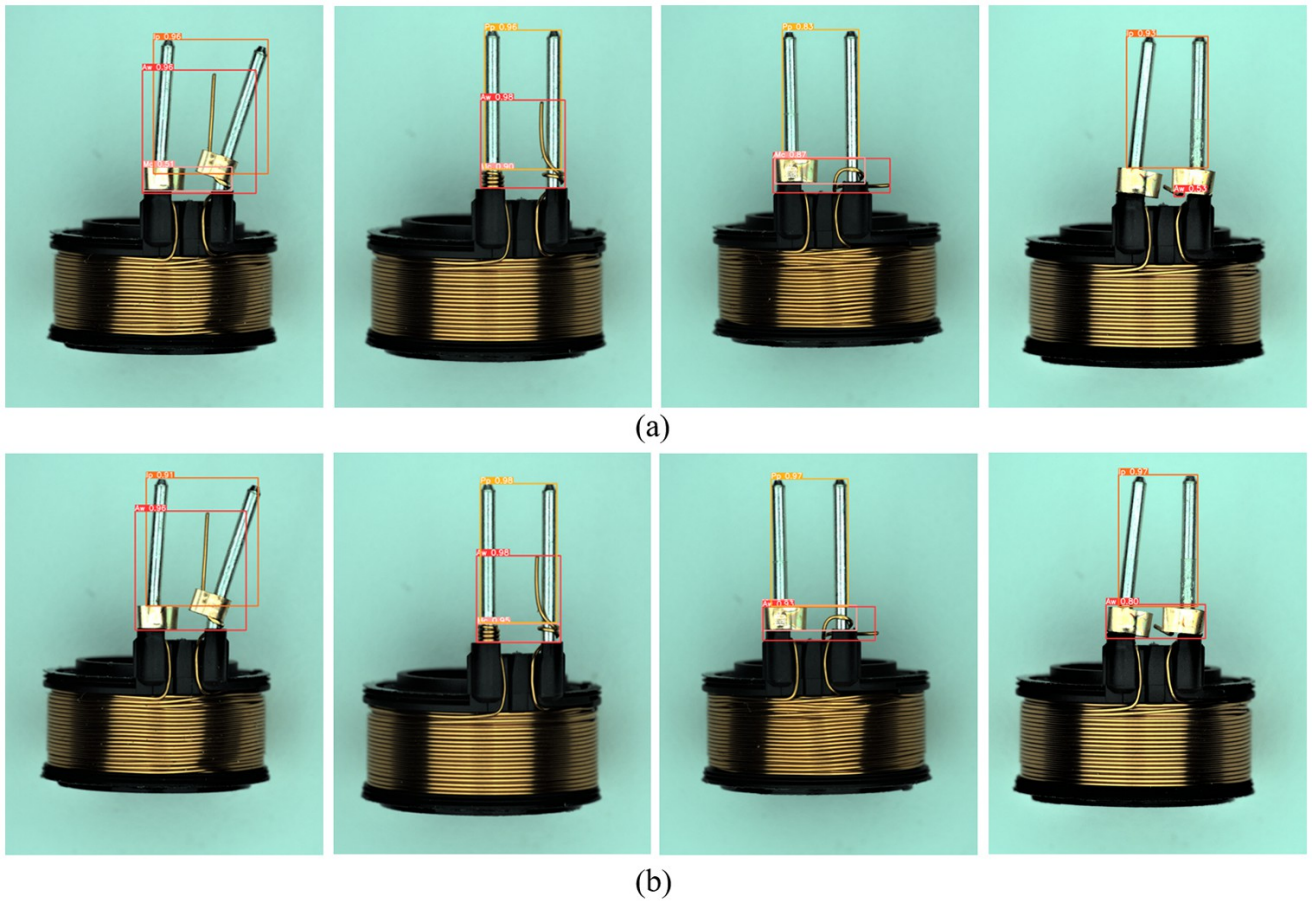
Visualization of detection results on solenoid connector dataset. (a) indicates the YOLOv5s detection results, (b) indicates the detection result of our proposed PO-YOLOv5.

We visualized and compared the proposed method with the original YOLOv5, as shown in Fig 9. It can be seen from the detection results that the original YOLOv5 model has low confidence in detecting inclined pins (Ip) and abnormal lines (Aw), even missing copper (Mc) defects are incorrectly detected. In contrast, our proposed PO-YOLOv5 improves the detection accuracy of tilted pin (Ip) and abnormal line (Aw) defects by adding a new prediction head that allows the network to learn richer semantic information. At the same time, the ODConv module was used to enhance the network’s ability to extract features, improving overall defect detection accuracy.

### Ablation study

In order to demonstrate the effectiveness of each improved component, we conducted ablation experiments. The results are shown in Tables 3 and 4. PO-YOLOv5 is the model proposed in this paper. It uses both the ODConv module and the added prediction head.

**Table 4:** Comparison of evaluation indicators for each mode.

Method	AP				mAP@0.5:0.95
	Mc	Aw	Ip	Pp	
YOLOv5s	81.4%	87.5%	82.1%	96.4%	87.1%
YOLOv5s+ODConv	81.9%	86.3%	83.1%	97.6%	87.6%
YOLOv5s+P6	83.2%	88.7%	85.4%	97.0%	88.5%
Ours	**84.1%**	**91.1%**	**86.5%**	**97.8%**	**90.1%**

Table 3 shows the ablation results for adding each component to the YOLOv5s model for training. The red numbers in the table show the improvement of each model metric relatively compared with the baseline model. The results show that the introduction of the ODConv module improves the mAP by 0.5% and barely affects the model size and inference speed, indicating that the ODConv module achieves a better trade-off between detection accuracy and detection speed. Adding a new prediction head to the YOLOv5s model increased volume slightly, but increased mAP 1.4% over the original YOLOv5s model, verifying the validity of the added prediction head. Compared with the original YOLOv5s, PO-YOLOv5 has a significant 3% increase in mAP on the Solenoid Connectors dataset, despite a slight growth in model size and parameters. In industrial defect detection, it is critical to improve detection accuracy while meeting the need of real-time detection. Therefore, a small increase in the parameters is worthwhile. Since the input image was resized to 1280×1280, the forward inference time of the model was increased, which reduced the FPS down to 62, but it still meets the requirements of real-time monitoring on industrial production lines.

Table 4 lists the mAP for each model in each detection category. As can be seen in Table 4, after adding the new prediction head, the detection accuracy of the model for defects containing relative position information, such as Inclined pin and abnormal wire are improved by 3.3% and 1.2%, respectively. It indicates that the addition of the new prediction head enables the model to obtain more semantic information and improves the model’s detection accuracy for defects containing relative position information. Moreover, the total detection accuracy is improved by replacing the conventional convolution with the ODConv module, which means that the ODConv module improves the feature extraction ability of the model. Finally, the proposed PO-YOLOv5 improved the detection accuracy for all categories, especially the detection accuracy of inclined pin defects has improved by 4.5%, which is a significant improvement.

To verify the dynamic convolution brings more improvement in the deep layer of the network than the shallow layer, we applied four ODConv modules to the Backbone and Neck, respectively, as shown in Table 5. The result shows that applying the dynamic convolution module to the deep layer of the network brings higher improvement than the shallow layer. We believe that stacking the ODConv modules in the deep layer of the network enables the model to extract more semantic feature information. In the following experiments, we will explore the optimal number of ODConv modules to stack in the neck.

**Table 5:** Performance of ODConv modules in different positions.

Method	Parameters	FLOPs	FPS	mAP@0.5:0.95
YOLOv5s	7.021M	15.8G	133	87.1%
YOLOv5s+ODConv(Backbone)	7.055M	12.1G	114	85.2%
YOLOv5s+ODConv(Neck)	7.052M	14.8G	122	87.6%

We tested the experimental results of stacking different numbers of ODConv modules in the Neck, which is shown in Table 6. As the number of ODConv modules rises, both the mAP and the parameters of the model increase. The model obtained the highest mAP when the conventional convolution was replaced with only four ODConv modules, indicating that the network had reached saturation. The four ODConv modules correspond to the four prediction heads of PO-YOLOv5. The feature information extracted from each layer of ODConv modules forms a multi-scale feature map after feature fusion and convolution operations, which improves the feature expression ability of the network. At this time, the detection effect of the model is optimal. Adding extra ODConv modules will reduce the accuracy of the model and increase the parameters. Unlike traditional convolution kernel parameters that are trained and shared by all samples, dynamic convolution assigns a weight factor to each convolution kernel depending on the input image, allowing the model to have a stronger representation ability. Therefore, as the number of ODConv modules increases, the parameters of the model also increase slightly.

**Table 6:** Ablation study on the number of ODConv modules.

Number of ODConv modules	Parameters	mAP@0.5	mAP@0.5:0.95	FPS
ODConv modules×1	12.349M	95.8%	88.0%	64.10
ODConv modules×2	12.362M	96.5%	88.7%	63.82
ODConv modules×3	12.369M	95.5%	88.1%	63.59
ODConv modules×4	12.380M	**96.8%**	**90.1%**	63.12
ODConv modules×5	12.405M	95.8%	88.7%	62.81
ODConv modules×6	12.452M	96.4%	88.2%	62.50

## Conclusion

In this paper, we proposed an improved model PO-YOLOv5 based on YOLOv5 to detect defects in solenoid connectors. With the addition of a prediction head, the semantic information extraction ability of the model was improved and it became more sensitive to defects with relative location information. The conventional convolution is replaced with ODConv modules in the Neck to improve the model representability. A dataset was built, containing images of defective solenoid connectors captured by the industrial camera and the ground truth labeled by hand. Furthermore, we proposed a labeling method based on relative position information, which was shown to have superior detection accuracy on the solenoid connector dataset. Experimental results have shown that the modified model improves the accuracy of recognizing defects in solenoid connectors, while ensuring real-time detection. Compared with the existing state-of-the-art detection models, the proposed model PO-YOLOv5 has better detection performance.

In the future, we will continue to optimize the solenoid connectors dataset and the method proposed in this paper. On the one hand, we will construct a larger dataset to enrich the diversity of defects and improve the detection accuracy. On the other hand, the PO-YOLOv5 method proposed in this paper has poor detection effect when detecting tiny targets. We will continue to investigate multi-scale detection methods to solve this problem. In addition, to detect solenoid connectors with different specifications and different manufacturing processes, we will explore the combination of transfer learning and YOLOv5 to make the model have better generalization performance in industrial applications.
